# Editorial: Pannexin and connexin signaling in cell death and tissue health and disease

**DOI:** 10.3389/fcell.2023.1264800

**Published:** 2023-08-02

**Authors:** Marc Mesnil, Andrew K. J. Boyce, Catherine S. Wright

**Affiliations:** ^1^ CoMeT Laboratory - UR 24344, University of Poitiers, Poitiers, France; ^2^ Department of Cell Biology & Anatomy, Cumming School of Medicine, University of Calgary, Calgary, AB, Canada; ^3^ Department of Biological and Biomedical Sciences, Glasgow Caledonian University, Glasgow, United Kingdom

**Keywords:** apoptosis, cell signaling, connexin, disease, gap junction, health, hemichannel, pannexin

This Research Topic of Frontiers in Cell and Developmental Biology, “*Pannexin and connexin signaling in cell death and tissue health and disease*,” brings together seven new articles exemplifying the role of connexin and pannexin signaling in human health, with the aim of increasing understanding of the biology of this area and feeding into work developing novel related therapies.

The first time that gap junctions were associated with a human disease was in 1966, when Loewenstein and Kanno observed a lack of gap junctional intercellular communication between liver cancer cells (Loewenstein and Kanno, 1966). During the following five decades, this association has been confirmed in many *in vitro* and *in vivo* models; however, there is yet no clear answer about the precise molecular involvement of gap junctions and their constitutive proteins, the connexins, in tumor progression. How connexins are involved in cancer cell growth and/or invasion is still an open question, whose possible answers depend on tumor stage and cancer type. The lack of clear molecular mechanisms linking connexin signaling to cancer is probably due to the complexity of the disease evolution and heterogeneity of its microenvironment (Aasen et al., 2019).

Since the seminal observation of Loewenstein and Kanno, it took almost 30 years to causally link connexin gene mutations to a human disease (in X-linked Charcot-Marie-Tooth disease) outside the cancer field (Bergoffen et al., 1993). This finding was unexpected, as the particular connexin, Connexin32 (Cx32), which was thought to be an epithelial-specific connexin, was identified in Schwann cells with a critical role in myelin maintenance. This work inspired the description of many more connexin-related genetic diseases, growing over 20 years, with the parallel discovery of new connexin isoforms, establishing connexins as members of a multigene family. Connexin mutations are now known to cause a wide range of genetic diseases affecting hearing (cochlea), skin keratinization, heart function, lens transparency, etc., (Srinivas et al., 2018). Many of these discoveries were confirmed by the increased use of transgenic strategies in mice permitting visualization of the pathophysiological effects of modulating connexin expression.

In parallel, investigations of connexin function and biology showed that at least some connexins could not only mediate direct intercellular communication through gap junctions, but also communicate between intracellular and extracellular domains through unopposed connexin channels (connexons) in the plasma membrane. This so-called hemichannel activity is associated with pathological events (ischemia, Ca^2+^ concentration modulation, inflammation, etc.). Subsequently, another family of large pore transmembrane proteins, the pannexins, was identified as the vertebrate homologs of innexins, gap junction proteins in invertebrates. It is now well established that pannexins indeed share similar functions with connexin hemichannels, particularly in cell death and inflammation by permitting the release of pro-inflammatory molecules (Makarenkova et al., 2018), but are unlikely to form gap junctions.

Our understanding of the involvement of connexins and pannexins in human health has rapidly expanded since 1966. This diversity appears in the seven articles gathered in this Research Topic. In this Research Topic, the implication of these proteins in the inflammatory cell response is largely presented in three reviews, with one extending to other large pore channels (innexins, calcium homeostasis modulator, and leucine-rich repeat-containing 8 proteins) including pannexin channels and connexin hemichannels (Vega et al.). Next, Pannexin1 (Panx1), one of the three pannexin family members, and its role in chronic and acute inflammation is specifically reviewed. Here, assuming Panx1 channel inhibition may improve outcomes for inflammatory diseases, the need for specific inhibitors as alternative therapeutics is emphasized, especially when vital organs are concerned (Rusiecka et al.). The importance of Panx1 activity in human health is confirmed in a further review by considering its various roles in diseases affecting the liver (Van Campenhout et al.). Finally, the importance of connexin hemichannel function is highlighted in bone tissue. Here, artificially increasing Cx43 hemichannel activity, known to increase prostaglandin E2 release under mechanical stimulation and thereby promote bone formation (Zhao et al.), is proposed as a mechanism to limit osteoporosis.

Apart from these four review articles, the three original articles of this Research Topic focus on three different connexins in various tissues. One of these connexins, the ortholog of human Cx36 (Cx35.1, abundant in electrical synapses) in zebra fish, is seen to be important for visual effects and related behavior, since its depletion causes hyperopia and visual-motor deficiencies (Brown-Panton et al.). Another article describes the consequences of Cx30.3 mutations affecting trafficking towards the plasma membrane, implicated in the rare skin disorder Erythrokeratodermia Variabilis et Progressiva (EKVP). Interestingly, as up to nine different types of connexins can be co-expressed in keratinocytes, this study shows that expression of other connexins permits transdominant rescue of Cx30.3, which can then be targeted to the plasma membrane. Such an astonishing effect is seen as a possible therapeutic strategy that should be investigated (Lucaciu et al.). The involvement of a third connexin (Cx26) in another skin disorder associated with hearing loss (Keratitis Ichthyosis Deafness syndrome) was explored through a mutation exhibiting hyperactive hemichannel activity in the supporting cells of the organ of Corti in the cochlea. By using appropriate *in vivo* and *in vitro* models, the authors deduce hyperactive Cx26 hemichannels induce cell damage by permitting Ca^2+^ influx, leading to hair (stereocilia) loss and subsequent deafness (Abbott et al.).

As we can see, the seven articles (four reviews and three original research articles) in this Research Topic show the complexity of pannexin and connexin involvement in tissue health and disease. While decades ago defective gap junctions were thought to be solely responsible for connexin-related human diseases, it now appears to frequently be a consequence of uncontrolled pannexin or connexin-mediated communication between the intracellular and extracellular space. A better understanding of the molecular mechanisms involved may permit connexins and pannexins to be targets for possible new therapeutic strategies.
